# Non-Image Forming Effects of Light on Brainwaves, Autonomic Nervous Activity, Fatigue, and Performance

**DOI:** 10.5334/jcr.167

**Published:** 2018-09-12

**Authors:** Taleb Askaripoor, Majid Motamedzadeh, Rostam Golmohammadi, Maryam Farhadian, Mohammad Babamiri, Mehdi Samavati

**Affiliations:** 1 Department of Occupational Health, School of Public Health, Hamadan University of Medical Sciences, IR; 2 Department of Ergonomics, School of Public Health and Research Center for Health Sciences, Hamadan University of Medical Sciences, IR; 3 Center of Excellence for Occupational Health, School of Public Health and Research Center for Health Sciences, Hamadan University of Medical Science, IR; 4 Department of Biostatistics, School of Public Health and Research Center for Health Sciences, Hamadan University of Medical Sciences, IR; 5 Department of Medical Physics and Biomedical Engineering and Research Center for Biomedical Technologies and Robotics (RCBTR), Tehran University of Medical Sciences, IR

**Keywords:** sleepiness, fatigue, light, EEG alpha activity, performance

## Abstract

Fatigue and sleepiness are one of the main causes of human errors and accidents in the workplace. The empirical evidence has approved that, in addition to stimulating the visual system, light elicits brain responses, which affect physiological and neurobehavioral human functions, known as the non-image forming (NIF) effects of light. As recent evidences have shown the positive effects of red or low correlated color temperature white light on alertness and performance, we investigated whether exposure to 2564 K light could improve subjective and objective measures of alertness and performance compared with 7343 K, 3730 K, and dim light (DL) conditions during the daytime. Twenty two healthy participants were exposed to the light while they were performing a sustained attention task and their electroencephalogram (EEG) and electrocardiogram (ECG) were recorded. Both 2564 K and 7343 K conditions significantly reduced EEG alpha-power compared with the DL and 3730 K conditions. Moreover, the 2564 K, 7343 K, and 3730 K conditions significantly reduced subjective fatigue, sleepiness and increased heart rate and performance compared with the DL condition. Furthermore, the effects of light conditions on alertness and performance varied over the day so that more effective responses were observed during the afternoon hours. These findings suggest that light interventions can be applied to improve daytime performance.

## Introduction

Many people work and spend most of their time in indoor environments [1]. Physical factors of such environments will affect the mood, motivation, and productivity of individuals [2,3]. Light is an essential and important physical factor that people work under specific lighting conditions in indoor environments [1]. Intrinsically retinal ganglion cells (ipRGCs) are a type of retinal photoreceptors that are particularly sensitive to blue light (460–480 nm). These cells send input signal to certain brain regions to express non-image forming (NIF) effects of light [4,5,6]. Besides the classic visual effects, light affects physiological processes such as melatonin suppression, circadian and homeostatic regulations, and neuroendocrine and neurobehavioral functions [7,8,9,10,11]. Furthermore, light can have acute physiological responses and positive effects on subjective and objective measures of alertness [12,13,14,15,16], mood [17], and certain task performance [18,19,20]. These effects are important because fatigue and sleepiness are major causes of human errors and accidents, and can reduce the workers’ efficiency [21,22]. However, few studies have assessed such effects of light in the workplace and everyday life.

A number of studies have shown that monochromatic blue light (short-wavelength) or high correlated color temperature (CCT) white light can increase alertness and performance during daytime [12,19,23,24,25,26,27]. However, recent evidence has shown the positive acute effects of red or low CCT light on alertness and performance during daytime and nighttime [14,15,28,29]. Furthermore, red light has a stronger effect than blue light during afternoon hours [14]. Because of color-salience, the use of monochrome light is not applicable in indoor environments [12]. In addition, most regular indoor lightings in everyday life settings are made up of white light that express different ranges of wavelength, brightness, CCT, and illumination level [30]. It is worth mentioning that the high proportions of long-wavelength (red) radiations in white light leads to a decrease in the CCT known as low CCT or warm white light. In contrast, the high proportions of short-wavelength (blue) radiations leads to an increase in the CCT known as high CCT or cool white light [15,26,31].

In an experimental study, Sahin et al. suggested that low CCT light increased alertness during daytime compared with dim light (DL) condition [15]. However, they did not compare the low and high CCT lights. Also, Baek and Min indicated that exposure to high CCT light significantly decrease alpha-band power and enhanced objective alertness and performance compared with white light during the afternoon hours [12], but the impact of low CCT light was not assessed in their study.

It is still unclear whether exposure to low CCT light (2564 K) during daytime under regular conditions (healthy day-active participants with regular sleep-wake cycle, and regular working hours) and conforming to the commonly experienced illumination level for the daytime work environments (500 lux on the desk) has positive impacts on fatigue, alertness, and performance [32,33]. The second question is whether time of day (morning vs. afternoon) can be a potential moderator of the effects of light. In order to answer these questions, in the present study, a simulated workplace-like environment was designed and the nature of routine daily tasks was also simulated by imposing an activity requiring attention and perception on the participants [12].

We hypothesized that the 2564 K, 3730 K, and 7343 K light conditions enhance alertness and performance compared with the DL (control) condition, and the 2564 K and 7343 K conditions improve alertness and performance compared with the 3730 K condition.

## Materials and methods

### Participants

Twenty-two healthy paid volunteers (male; mean ± SD age: 27.32 ± 3.63 years) were studied. All participants were nonsmokers, did not have any major health problem, including mental and physical illnesses, and were not taking any medication. In addition, the participants had no history of eye diseases and, based on the Ishihara test, they all had normal color vision (Kanehara Shupman Co., Tokyo, Japan). The participants who had a history of shift-work or travel to a different time zone during the three months prior to the experiment and those who were diagnosed as extreme early or extreme late chronotypes, based on the results of the Munich Chronotype Questionnaire [34], were excluded from the study. The participants’ sleep quality was assessed using the Pittsburgh sleep quality index (PSQI). To ensure the maximum circadian-phase homogeneity and to control the potential differences in fatigue and sleepiness levels due to circadian variations and sleep pressure at different light conditions, only volunteers with a good sleep quality (PSQI score <5) [35] and regular sleep-wake state (bedtimes between 22:00 and 24:00 and wake up between 07:00 and 08:00) were enrolled into the study. In addition, the participants filled out a sleep/wake log, starting one week prior to the experiment and were asked to keep a regular sleep/wake state during the study. The ethics committee of Hamadan University of Medical Sciences approved all the study materials and procedures, and the participants signed an informed consent before the commencement of the study.

### Study design and procedures

The study had a repeated-measures design and each participant was exposed to four light conditions in a counterbalanced order at one-week intervals. They were asked to arrive at the experimental facility at 07:45 for four consecutive weeks. As a preparation period, they were kept seated in a dim room (<5 lux at eye level from a 3520 K fluorescent lamp) for about 45 minutes at the time of arrival. During this period (and also between 13:05 and 13:50), the participants received instructions and were fitted with electrocardiogram (ECG) and electroencephalogram (EEG) electrodes. Morning and afternoon sessions started at 8:30 and 13:50 respectively, and lasted approximately 92 min.

Each participant was first exposed to a 13-min period of dim light (<5 lux at eye level from a 3520 K fluorescent lamp, baseline) followed by a different light condition each week: a DL condition (<5 lux at eye level from a 3520 K fluorescent lamp), or 500 lux illuminance level on the desk from the 7343 K, 2564 K, or 3730 K light condition. Notably, the light settings (500 lux) were selected in keeping with the norms of daytime office environments [32,33].

**Figure 1:**
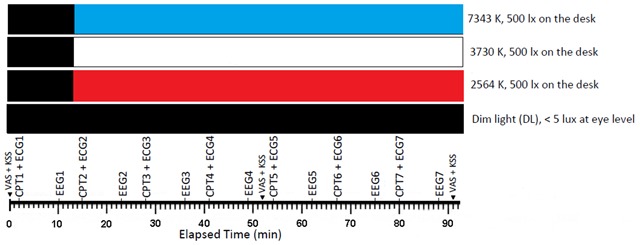
The study setup. The protocol included continuous performance test (CPT), electroencephalogram (EEG), electrocardiogram (ECG), Karolinska Sleepiness Scale (KSS), and visual analogue scale (VAS) for fatigue and sleepiness. EEG, CPT1, and ECG1 indicated that the data acquired during the initial dim period has been used as a baseline for normalization.

In each session (morning vs. afternoon), seven repeated sequences (trials) that comprised of a continuous performance test (CPT) and an ECG (~8 min) followed by an EEG (3 min) were performed. The Visual Analogue Scale (VAS) for fatigue and Karolinska Sleepiness Scale (KSS) were rated by the participants three times (at the beginning of the experiment, at 52th minute, and at end of the experiment). The experimental design is summarized in Figure 1. During the breaks between the sessions, the participants were seated in the dim room (<5 lux at eye level) and kept themselves busy by talking, drinking, and working on a computer with a dimmed monitor. The breaks were similar across all experimental conditions. Light meals were provided between 12:00 and 12:30 for the participants.

Since routine daily activities usually require constant alertness with the minimum mental effort to remain alert, to create a realistic condition, a sustained attention task (continuous performance test) was imposed on the participants [12]. Since the EEG alpha activity is associated with sustained attention task [12,36], we focused our analysis on alpha power recorded under the four light conditions.

### Lighting settings

The study was performed in an air-conditioned environment (dimensions: 3.4 × 5.6 × 3.05 m). The main furnishings of the room consisted of a desk with computer and chair. The room’s windows were covered with light-blocking curtains to prevent penetration of daylight into the experimental environment. The participants sat behind a desk most of the time making their position almost fixed in the experimental setting. They were also asked to keep their eyes open during the experiment.

The experimental room was illuminated with dimmable fluorescent light sources installed on the ceiling, including seven ceiling tubes (Philips, MASTER TL-D Super 80 36 W/840 and 36 W/827, CCT = 3730 K and 2564 K), and nine ceiling tubes (Osram, sky white 36 W/880, CCT = 7343 K). Each group of the light sources had a separate circuit and a control switch. Commercially available light sources were used to create a realistic experimental lighting ambiance. The horizontal illuminance level (0.75 m at the desk) was measured using an illuminance meter (Hagner, model E2, Solna, Sweden). The mean horizontal illuminance level was 519 lx (SD = 9.2 lux). The measured illuminance uniformity (E_min_/E_ave_) was > 0.8 indicating the homogeneity of illuminance in the experiment environment and none of the lighting sources were in the direct view of the participants.

**Figure 2:**
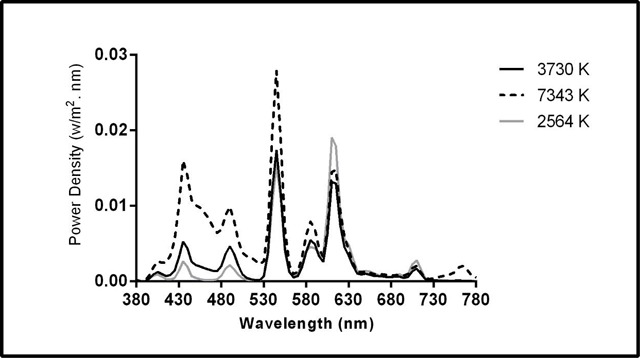
Spectral power distribution measured at eye level in the 7343 K, 2564 K, and 3730 K light conditions.

The illuminance level, CCT, Color Rendering Index (CRI), and chromaticity coordinates were measured at eye level (1.2 m from the floor) using a spectrometer (C7000 SpectroMaster, Sekonic Corp., Tokyo, Japan). Table 1 presents the photometric values at eye level. It is worth noting that the supplementary materials of Lucas et al. (2014) were used to calculate the photometric values [37]. Figure 2 shows the spectral power distribution of the light conditions.

**Table 1:** Photometric values measured at eye level for the 7343 K, 2564 K, and 3730 K light conditions.

Variable	7343 K	3730 K	2564 K

Color Rendering Index (CRI Ra)	86.9	82.9	81.9
Chromaticity coordinates (x,y) 1931 CIE chromaticity coordinates	0.3023, 0.3117	0.3950, 0.3899	0.4831, 0.4327
Photopic illuminance (lux)	333	332	333
Melanopic lux (α-opic lux)	342	195	115
Cyanopic lux (α-opic lux)	414	180	73
Chloropic lux (α-opic lux)	340	291	259
Rhodopic lux (α-opic lux)	349	236	169
Erythropic lux (α-opic lux)	323	319	326
Photon density (photons.cm^–2^.s^–1^)	3.14 × 10^14^	2.7 × 10^14^	2.7 × 10^14^
Irradiance (μW.cm^–2^)	117	96	92

Notably, the illuminance level coming from the monitor screen during the CPT or break between sessions was less than 2 lx at eye level, lower than the threshold for inducing an alerting response and affecting the nervous system [38,39]. This was constant in all experimental conditions. It is important to note that all the light condition spectra, i.e. illuminance level (500 lux on the desk) and CRI (CRI ≥ 80) were in agreement with the lighting standards (EN 2011) for indoor environment such as offices.

### Electroencephalogram

EEG data were recorded using an EEG analyzer, with Ag/AgCl electrodes (Encephalon131–03, Medicom MTD, Russia). Following the international 10–20 system, the EEG data were collected from the Z-line on the participant’s scalps at positions of Oz, Pz, Cz, and Fz [40]. The ground electrode was placed on the forehead and the reference electrodes were placed on the left and right earlobes (A1 and A2). To monitor eye blinks, one electrode was located directly below the right eye. The data on EEG were recorded at 250 Hz (band-pass filter, 0.3–40 Hz) and its impedance was kept below 5 KΩ. In order to minimize EEG artifacts, the participants were asked to sit quietly, keep their eyes open, fix their gazes at an X point printed on the opposite wall, and remain still without moving, talking, or blinking during EEG recording trials. The average value of A1 and A2 channels was calculated and subtracted from the other channels. The data were grouped into 2-second (s) epochs with 1 s overlap from each epoch. The epochs with an artifact of noise, body movements, and eye blinks were excluded from the analysis. Then, a 10% cosine window followed by a fast Fourier transform were applied for each epoch. This process yielded spectral power distributions from 0.3 to 40 Hz at 0.5 Hz intervals. Afterwards, the average power from each epoch was calculated to achieve the average power for each trial. The alpha frequency range (8–13 Hz) was selected for analysis and the average powers derived from the Fz, Cz, Pz, and Oz channels were used for the calculation of the alpha power. In order to compare EEG results, the EEG power was normalized by the power acquired during the initial dim time (EEG1). The MATLAB software package (ver. R2012a, Math-Works, USA) was used to analyze the EEG data.

### Continuous performance test (CPT)

The CPT test was performed through displaying a randomly-varying stimuli (single numbers between 0–9) on a black monitor screen for 500 ms with a 1000 ms interval [41]. The participants were asked to react by pressing the space bar button as soon as the target stimulus (number ‘4’) was appeared. To avoid learning effect, a randomized stimulus was used for each experimental trial. Furthermore, to minimize the difference in learning the task between the participants, a sequence of practice trials were performed one day before the experiment. The CPT data were normalized by the data acquired at the initial dim time (CPT1). During the data analyses, the normalized mean reaction time (RT) of the correct responses and the accuracy were used as dependent measures.

### Heart rate and heart rate variability

Heart rate (HR) and heart rate variability (HRV) were measured using “HRV” software analysis (Medicom MTD, Russia). The ECG electrodes were placed at V1 and V6 locations using single-use electrodes (Kendall, H124SG, Germany). The ground electrode was the one used for the EEG recordings and it was placed on the forehead. The ECG was recorded at 250 Hz sampling rate and impedance was kept below 5 KΩ. The mean heart rate (bpm), LF (0.04–0.15 Hz), HF (0.15–0.40 Hz), and the LF/HF power ratio were used. The ECG parameters at each interval were normalized by the power obtained during the initial dim period (ECG1).

### Visual Analog Scale for fatigue

The subjective fatigue (subjective feeling of tiredness) was measured using an 11-point visual analog scale, ranging from zero for ‘completely not tired’ to 100 for ‘very tired’ [17,42].

### Karolinska Sleepiness Scale (KSS)

The subjective sleepiness of participants was assessed with the KSS [43] ranking response of 1 to 9, where 1 = “extremely alert”, 3 = “alert”, 5 = “neither alert nor sleepy”, 7 = “sleepy”, and 9 = “very sleepy. The VAS and KSS scores were normalized by the score acquired during the initial dim period.

### Statistical analysis

Data were analyzed using repeated-measures analyses of variance (ANOVA). When necessary, the Greenhouse–Geisser adjustment was applied. The ANOVA was performed for the four light conditions × six trials to analyze the data of alpha power, HR, HRV parameters, and CPT. Moreover, the ANOVA was performed for four light conditions × two trials to assess the subjective fatigue and sleepiness. Two-tailed paired Student’s t-tests with Bonferroni correction (when appropriate) were applied for further comparisons. Values are reported as means ± standard error. The data were analyzed using version 20.0 of the SPSS software (IBM, Armonk, NY, USA) and a p-value < 0.05 was considered statistically significant.

## Results

Table 2 presents the ANOVA results for EEG-alpha, HR, HRV measures, fatigue, sleepiness, and CPT. Table 3 shows the results of the two-tailed paired Student’s t-tests in the morning and afternoon sessions.

**Table 2:** Analyses of Variance (ANOVA) results for EEG- alpha, HR, HRV measures, fatigue and sleepiness, and CPT.

	*df*	*Error*	*F*	*p*

*EEG- alpha*				

Light	2.138	44.894	28.266	**<.001**
Session	1	21	3.649	.070
Trial	2.797	58.74	72.806	**<.001**
Light × Session	3	63	3.949	**.015**
Light × Trial	4.774	100.26	7.901	**<.001**
Session × Trial	3.115	65.419	.574	.72
Light × Session × Trial	5.499	115.472	1.706	.132
*HR*				

Light	3	63	10.256	**<.001**
Session	1	21	5.507	**.026**
Trial	5	105	.295	.915
Light × Session	2.064	43.351	.337	.723
Light × Trial	6.626	139.153	.398	.894
Session × Trial	5	105	.292	.916
Light × Session × Trial	7.337	154.078	0.633	.736
*LF*				

Light	2.07	43.468	7.428	**.002**
Session	1	21	.145	.707
Trial	2.133	44.802	4.792	**.012**
Light × Session	2.043	42.908	.234	.797
Light × Trial	6.357	133.491	.768	.603
Session × Trial	2.604	54.68	2.263	.1
Light × Session × Trial	5.453	114.521	1.261	.283
*HF*				

Light	1.843	38.709	.444	.629
Session	1	21	2.039	.168
Trial	2.316	48.627	9.84	**<.001**
Light × Session	2.216	46.531	.071	.946
Light × Trial	3.932	82.575	1.2	.317
Session × Trial	2.264	47.553	.482	.644
Light × Session × Trial	5.713	119.977	1.333	.25
*LF/HF*				

Light	1.831	38.451	5.636	**.009**
Session	1	21	.508	.484
Trial	2.544	53.421	2.315	.096
Light × Session	1.924	40.414	.542	.579
Light × Trial	4.294	90.177	1.547	.192
Session × Trial	2.834	59.515	.975	.407
Light × Session × Trial	5.605	117.695	2.277	**.045**
*Subjective fatigue*				

Light	3	63	12.649	**<.001**
Session	1	21	16.483	**.001**
Trial	1	21	45.017	**<.001**
Light × Session	3	63	.547	.652
Light × Trial	3	63	12.045	**<.001**
Session × Trial	1	21	3.141	.091
Light × Session × Trial	3	63	1.294	.284
*Subjective sleepiness*				

Light	3	63	20.632	**<.001**
Session	1	21	3.012	.097
Trial	1	21	9.991	**.005**
Light × Session	3	63	.2	.896
Light × Trial	3	63	5.552	**.002**
Session × Trial	1	21	13.975	**.001**
Light × Session × Trial	3	63	3.451	**.022**
*CPT mean RT*				

Light	3	63	17.282	**<.001**
Session	1	21	11.26	**.003**
Trial	1.548	32.51	1.303	.279
Light × Session	3	63	5.185	**.003**
Light × Trial	3.856	80.972	1.795	.14
Session × Trial	2.536	53.258	1.062	.365
Light × Session × Trial	5.2	109.201	1.577	.17
*CPT accuracy*				

Light	3	63	.497	.686
Session	1	21	1.072	.312
Trial	5	105	48.146	**<.001**
Light × Session	3	63	.664	.577
Light × Trial	7.705	161.806	2.008	.051
Session × Trial	4.482	94.126	1.192	.32
Light × Session × Trial	7.876	165.405	.307	.961

*Note*. Significant differences (p < .05) are shown in bold.

**Table 3:** T-test results for EEG-alpha measure, mean RT of CPT, HR, HRV measures, fatigue, and sleepiness during each session.

*Pairs*	*t*	*p*

**Session 1**		

*EEG-alpha*		

7343 K – DL	–5.157	<.001
2564 K – DL	–3.670	.001
*CPT reaction time*		

3730 K – DL	–3.229	.004
7343 K – DL	–4.053	.001
2564 K – DL	–3.817	.001
*HR*		

7343 K – DL	3.253	.004
2564 K – DL	3.862	.004
*LF/HF*		

2564 K – DL	–3.232	.004
*Subjective fatigue*		

7343 K – DL	–3.893	.001
2564 K – DL	–3.792	.001
*Subjective sleepiness*		

7343 K – DL	–5.694	<.001
2564 K – DL	–4.557	<.001
**Session 2**		

*EEG-alpha*		

3730 K – DL	–3.255	.004
7343 K – DL	–8.339	<.001
2564 K – DL	–6.485	<.001
3730 K – 7343 K	6.366	<.001
3730 K – 2564 K	4.172	<.001
*CPT mean RT*		

7343 K – DL	–6.014	<.001
2564 K – DL	–6.074	<.001
7343 K – 3730 K	–3.029	.006
*HR*		

3730 K – DL	4.372	<.001
7343 K – DL	4.428	<.001
2564 K – DL	3.619	.002
*Subjective fatigue*		

7343 K – DL	–4.302	<.001
2564 K – DL	–3.223	.004
*Subjective sleepiness*		

3730 K – DL	–3.111	.005
7343 K – DL	–5.936	<.001
2564 K – DL	–5.905	<.001

*Note*. Only comparisons that were statistically significant are shown in the table. Bonferroni correction was applied and p ≤ 0.006 was set as statistically significant.

### EEG

**Figure 3:**
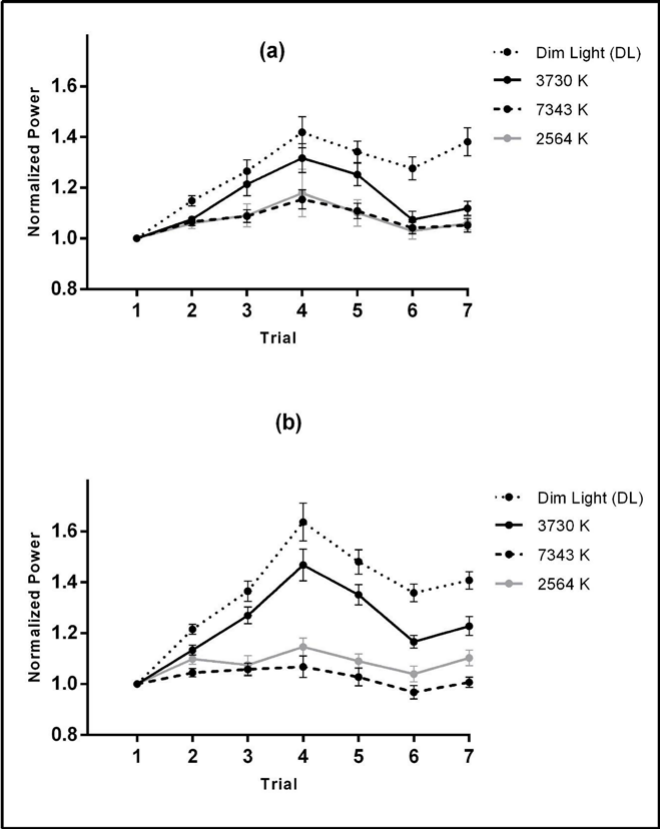
Means ± SE of normalized alpha power for 3730 K, 7343 K, 2564 K, and DL conditions in each trial for morning **(a)** and afternoon **(b)** sessions. *Note:* Given that the alpha power was negatively correlated with light-induced alertness, the increment of alpha power indicates a reduction in objective alertness [14,15,44,45].

Two-tailed paired Student’s t-tests with Bonferroni corrections revealed that the alpha power after exposure to 2564 K [1.09 ± 0.026; t = –5.496, p < 0.001], 7343 K [1.057 ± 0.016; t = –8.286, p < 0.001], and 3730 K [1.222 ± 0.023; t = –4.291, p < 0.001] was significantly lower than that after exposure to the DL condition [1.358 ± 0.031]. Moreover, 7343 K [t = –6.517, p < 0.001] and 2564 K [t = –3.283, p = .004] had significant differences with 3730 K condition. The normalized alpha power was significantly higher during the session 2 as compared with the session 1 indicating that the participants were less alert during the session 2 as compared with the session 1. Figure 3 shows the normalized alpha power for each trial in each session.

### CPT (performance data)

**Figure 4:**
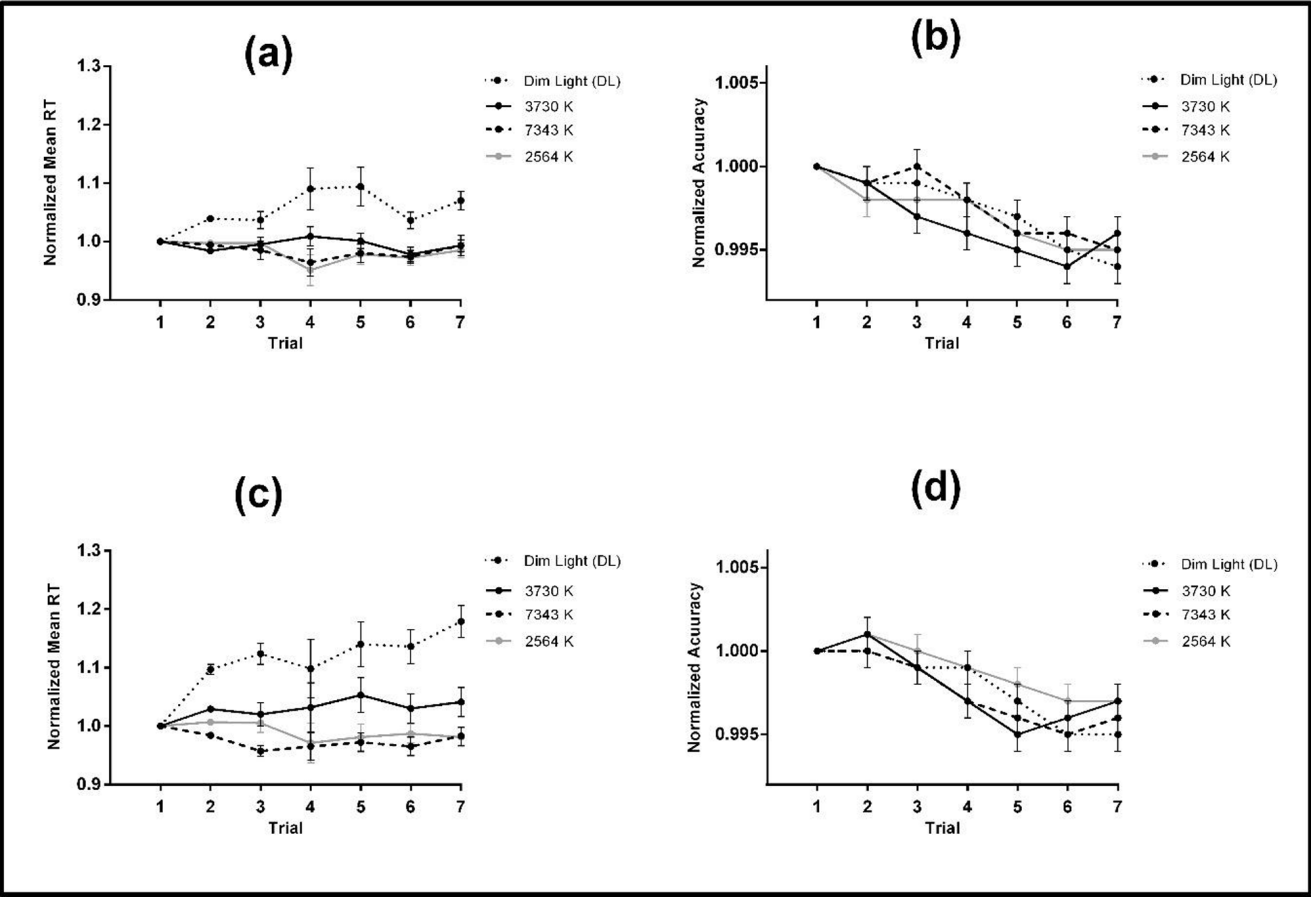
Means ± SE of the normalized mean RT and accuracy for the 3730 K, 7343 K, 2564 K, and DL conditions in each trial for morning (**a** and **b**) and afternoon (**c** and **d**) sessions.

The normalized mean RT were significantly lower under 2564 K [0.984 ± 0.012; t = –5.447, p < 0.001], 3730 K [1.014 ± 0.012; t = –3.364, p = 0.003], and 7343 K [0.976 ± 0.01; t = –5.918, p < 0.001] conditions as compared with the DL [1.095 ± 0.019] condition. The other comparisons did not indicate significant differences. The mean RT was significantly higher during the afternoon session than the morning session showing that the participant’s performance was decreased during the afternoon in comparison with the morning session. Generally, the accuracy was reduced over the experiment. Figure 4 shows the mean RT and accuracy of CPT task in each trial for each session.

### Heart rate (HR) and heart rate variability (HRV)

**Figure 5:**
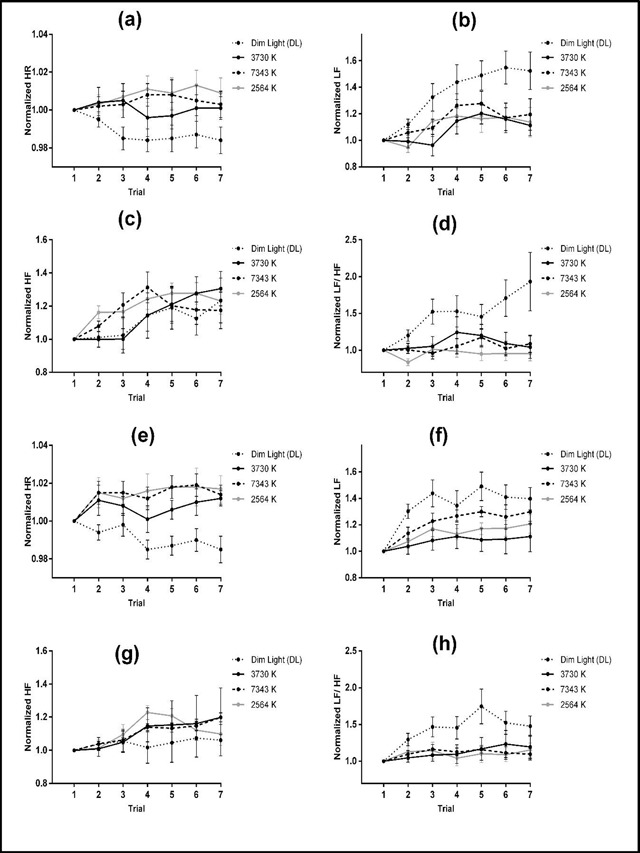
Means ± SE of the normalized HR, LF, HF and LF/HF power ratio for the 3730 K, 7343 K, 2564 K, and DL conditions in each trial for the morning (**a, b, c,** and **d**) and afternoon (**e, f, g,** and **h**) sessions.

Post hoc t-tests indicated significant differences between the 2564 K [1.012 ± 0.004; t = 4.857, p < 0.001], 3730 K [1.004 ± 0.003; t = 3.506, p = 0.002], and 7343 K [1.01 ± 0.004; t = 4.496, p < 0.001] with DL [0.988 ± 0.003] condition in terms of HR measure. The other comparisons did not show significant differences. Significant differences between 3730 K [1.091 ± 0.044; t = –3.808, p = 0.001] and 2564 K [1.14 ± 0.043; t = –3.207, p = 0.004] conditions with the DL condition were observed [1.402 ± 0.069] in terms of LF measure. The remaining comparisons did not show any significant differences. No significant difference was observed between the light conditions in terms of HF measure. In addition, significant difference between the 2564 K [1.03 ± 0.054; t = –3.516, p = 0.002] and the DL [1.53 ± 0.134] conditions was found in terms of normalized LF/HF power ratio. The other comparisons did not indicate any significant differences. Figure 5 shows the normalized HR, LF, HF, and LF/HF power ratio in each trial for each session.

### Subjective Fatigue

The participants felt less tired under the 3730 K [1.215 ± 0.028; t = –3.372, p = 0.003], 2564 K [1.151 ± 0.032; t = –4.453, p < 0.001], and 7343 K [1.13 ± 0.029; t = –5.914, p < 0.001] conditions compared with the DL [1.376 ± 0.036] condition. The other comparisons did not indicate significant differences. Furthermore, they felt more tired during the afternoon session than the morning session and during the trial 2 than the trial 1.

### Subjective sleepiness

The participants felt lower sleepiness under the 3730 K [1.27 ± 0.056; t = –3.403, p = 0.003], 2564 K [1.161 ± 0.049; t = –6.753, p < 0.001], and 7343 K [1.107 ± 0.036; t = –7.614, p < 0.001] conditions than the DL [1.519 ± 0.052] condition. The other comparisons did not show any significant differences. In general, the participants felt sleepier during the afternoon session in comparison with the morning session and during the trial 2 than the trial 1. The means ± SE of the normalized subjective fatigue and sleepiness during each session are presented in Table 4.

**Table 4:** Means ± SE of the normalized subjective fatigue and sleepiness during each session.

Light Condition	Session 1	Session 2

*subjective fatigue*		

3730 K	1.153 ± 0.031	1.277 ± 0.044
7343 K	1.116 ± 0.036	1.145 ± 0.048
2564 K	1.086 ± 0.039	1.215 ± 0.048
DL	1.321 ± 0.042	1.43 ± 0.053
*subjective sleepiness*		

3730 K	1.178 ± 0.056	1.363 ± 0.096
7343 K	1.043 ± 0.041	1.17 ± 0.071
2564 K	1.098 ± 0.049	1.223 ± 0.085
DL	1.443 ± 0.068	1.595 ± 0.077

## Discussion

Our results showed that both 7343 K and 2564 K conditions significantly reduced alpha-power as compared with the DL and 3730 K conditions. Moreover, participants had a significantly lower reaction time in 7343 K and 2564 K compared with DL condition. Comparing with 3730 K, the participants had a better reaction time at 7343 K and 2564 K conditions. However, the differences were not statistically significant. Importantly, alpha power was negatively correlated with objective alertness [14,15,44,45]. Thus, reductions in alpha power were accompanied by an enhancement in alertness. These findings are consistent with earlier studies that indicated both blue and red lights had alerting effects at night and day time [28,46,47]. Field studies [25,26,48] have shown that 17000 K light condition decreased fatigue and sleepiness and improved the performance during daytime. Sahin et al. indicated that 2568 K (361 lx at eye level) light condition decreased alpha-power and enhanced objective alertness compared with a DL (<5 lux at eye level) condition during daytime [15].

In the present study, the 7343 K and 2564 K conditions decreased alpha-power, subjective fatigue, and sleepiness during the morning session as compared with the DL condition. However, no significant improvements were observed in the participants’ alertness and performance in comparison with the 3730 K condition that is generally used in working environments. These findings are in accordance with those of Smolders and de Kort [33] who reported no significant difference in performance and sleepiness between 2700 and 6000 K light (500 lx on the desk) conditions in the morning time. In addition, the findings of Sahin et al. study indicated that 2568 K light condition did not lead to an enhancement in the objective and subjective measures of alertness and performance during the morning hours [15].

The non-significant difference in the participants’ alertness and performance between 7343 K and 2564 K compared with the 3730 K condition during the morning session (Table 3) can be explained by the fact that the impact of light intervention can be more easily seen when human fatigue and sleep pressure are high (i.e., at night time) [44]. In addition, fatigue and sleepiness are affected by an interaction between the homeostatic need for sleep and circadian rhythm [49,50] and under regular condition, homeostatic sleep is low in the morning hours. In addition, in the present study, only participants without sleep deprivation and with a regular sleep-wake cycle were studied.

In contrast, in the afternoon session, the alpha power was significantly decreased under the 7343 K and 2564 K conditions as compared with the DL and 3730 K conditions (Table 3). Sahin et al. reported that 2568 K light condition was more effective in increasing the alertness during the midafternoon hours than DL condition [15]. In addition, Baek and Min showed that alertness and performance were significantly increased under high CCT light condition during the afternoon (post-lunch dip) as compared with white light condition [12].

Overall, the fatigue and sleep pressure increase with increasing waking hours during daytime [14]. Most people experience a significant increase in sleep pressure and fatigue during the midafternoon hours (between 14:00 and 16:00 h) that is called post-lunch dip [51]. The circadian drive is not sufficiently strong to counteract the increased sleepiness during this specific time [52,53]. In the present study, the observed more effective effects on allertness during the afternoon session are consistent with earlier studies reporting that light induced more alertness response when homeostatic sleep pressure increased [54,55]. It is important to emphasize that the increased sleep pressure in the afternoon (post-lunch dip) can lead to increase human errors, accidents, and reduce performance [22]. Thus, light interventions can disrupt this decline in allertness and improve individuals’ safety and performance.

In the present study, the volunteers had regular sleep-wake state and they were asked to keep a regular 8-h sleep-wake cycle throughout the study period. In addition, all participants met the criteria of average sleep durations. However, we did not use an objective measure (e.g. actigraphy) to confirm these self-reported data. Therefore, potential differences in fatigue and sleepiness due to circadian variations and sleep pressure may affect the observed results of the light conditions on the participants’ alertness in each session. Thus, these findings should be interpreted with care.

Smolder et al. indicated that bright light (1000 lx at eye level) modulated brain activity and enhanced alertness during the afternoon [56]. Also, Min et al. reported that bright light (700 lx at eye level) decreased alpha activity and increased alertness during the daytime. However, this light condition has led to a worsening reaction time and performance due to the high brightness and glare during a sustained attention task [45]. Glare must be accounted for creating effective lighting setup in real-life settings. Based on the results of the present study and positive effects of the 2564 K and 7343 K conditions on alertness, particularly in the afternoon, it can be concluded that altering the spectrum of light such as the use of high or low correlated color temperature lights may be more practical to affect the human neurobehavioral functions compared with bright light condition in working environments.

Neurophysiological evidence supports our findings about the relatively better effectiveness of the 2564 K and 7343 K conditions in improving alertness and performance compared with the 3730 K and DL conditions. As mentioned in the introduction, the available evidence shows that besides rods and cones, ipRGCs are particularly sensitive to blue light [4,5] expressing the non-imaging forming effect of light such as regulation of circadian system, melatonin suppression, neuroendocrine, and neurobehavioral functions [6,7,8,9,10].

It is well accepted that melatonin suppression is required to affect alertness at night [57,58]. However, recent studies have shown that melatonin level is low during the daytime, and light-induced alertness is not always mediated by the melatonin suppression [14,17,59]. Studies that have investigated the improvement of the level of the alertness by red light or warm white light have suggested that such an effect might be attributed to other types of photoreceptors (i.e., ipRGCs, cones and rods) [14,15]. Moreover, some studies suggested that warm colors such as red, yellow, and orange increase feelings of arousal and excitation that affect brain activity and motor responses and improve the alertness and performance [60,61]. Overall, it can be said that different mechanisms are mediated the physiological and neurobehavioral effects of light.

In the present study, the participants felt sleepier and more tired during the afternoon as compared with the morning session and during the trial 2 than the trial 1. These findings are consistent with our expectations and the results of previous studies that indicated participants become sleepier in the afternoon [15] and as time passes within each session [29]. It should be noted that the increased fatigue and reduction of the participants’ alertness and performance in the afternoon may be related, to some extent, to the study design. The participants were sited in a DL room between the sessions that may intensify the homeostatic sleep pressure in the afternoon.

In the present study, the 2564 K, 7343 K, and 3730 K conditions increased HR compared with the DL condition. This finding is consistent with the earlier studies reporting that bright light increased HR in comparison with DL in humans [62,63,64,65]. The 2564 K condition decreased HRV measure (LF/HF power ratio) compared with the DL condition. Canazei et al. (2016) reported that increase in the proportion of long wavelengths in the polychromatic white light led to a decrement in LF/HF power ratio during the nighttime [66]. In contrast, Smolders et al. found that CCT had no significant effect on HR and HRV (LF/HF ratio) during the daytime [33]. This discrepancy may be attributed to the fact that we investigated different CCT, light intensity, and photic inputs as compared with the Smolders et al. study. In addition, the effects of light on HR and HRV measures may depend on other variables such as time of exposure and type of activities performed by the participants [13,64].

The present study had some limitations. First, although we selected volunteers with regular sleep-wake state and asked them to keep it, we did not use an objective method (e.g. actigraphy) to validate these self-reported data. Second, the participants were relatively young and given the potential changes in the retina and neural structures of the eyes with age, the results of the current study cannot be generalized to older groups of people. Third, the duration of light exposure was relatively short (i.e., 92 min in each session) and we did not measure alertness and fatigue after the offset of the lighting. Therefore, it cannot be predicted whether the effects would persist throughout the day. In addition, the relatively long break between the sessions and the applications of DL condition during the break and the adaptation period before the actual light manipulation might lower the generalizability of the present results to a real working environments. Further studies are recommended to investigate older groups of people and actual workplace environments.

## Conclusion

Both 7343 K and 2564 K light conditions had strong stimulating effects in modulating the brain activities and had positive effects on sleepiness, fatigue, and performance, as compared with the 3730 K and DL conditions during the daytime. Furthermore, the effects of light conditions on sleepiness and performance varied over the day and light had a more effective response during the afternoon hours. The study provides further evidence in favor of the sensitivity of the autonomic nervous activity (e.g., HR and HRV parameters) to the light spectra (i.e., CCT) during the daytime. Light interventions can be useful in improving public well-being and safety, increasing the quality of indoor environments and the productivity of people working in such environments, and enhancing students’ ability to learn at school and other possible chronobiological applications. Thus, further field studies are recommended.
